# John Banister: The Troubles of a Seventeenth Century Oculist

**Published:** 1906-05-19

**Authors:** 


### John Banister: The Troubles of a Seventeenth Century Oculist.

We are accustomed to think of oculists in the seventeenth and eighteenth centuries as quack doctors, and to place them in the same class as the cures for hernia and the cutters for the stone. Many indeed were itinerant quacks who couched a cataract in a country town, and then moved on before the results of the operation had become manifest in the loss of the patient's eye; and such practitioners always took the fee in advance. But from time to time a real ophthalmic surgeon would arise, and would endeavour to put the practice of his profession upon a sound basis. Such a one was Richard Banister, Master in Surgery, oculist and practitioner in physick. He practised at Stamford in the reigns of Queen Elizabeth and James I., dying about 1624. He was educated by his illustrious kinsman, John Banister, a gentleman, and one of the best-known surgeons in London during the latter part of Elizabeth's reign. Richard says of himself that "my special breeding hath been in the general skill of Chirurgerie, but the desire I bear to be beneficial to the Commonwealth and the deare esteem I had of the health of my countrymen together with my care to bring credit to the Art of Chirurgerie was the cause that within a few years I left the greatest mass of that unmeasurable mysterie, as a heap too heavy for my undergoing to take up only some particular pieces wherein I might the better proceed to some perfection. Since I perceive what all confess that the Art of Chirurgerie is a sea unsounded and seeing also that it is manifestly remarkable that the most exquisite Chirurgian excelleth but in some few points of his profession: why should they not content themselves to profess no more than they excel in. Considering then these things I thought the art would be no loser by me, if I did let go many parts of her to hold the rest more sure and certain. And finding some defects in mine own eyes I choose their cure for my care that so I might benefit myself first and others after by mine own experience: unto this also I adjoyned the helpe of hearing by the instrument, the cure of the Harelip and the Wryneck. When in the threshold of my practice I could couch the Cataract and so begin to gaine some name of an Oculist, I laboured to advance my skill by the advice of the most skilful in those times: as namely Henry Blackborne, famous for the forenamed cures: my kind acquaintance Robert Hall of Worcester: Master Velder of Fennie Stanton: Master Surflet of Lynne and Master Barnabie of Peterborough, all excelling in these operations: and by familiar fellowship with them I grew into acquaintance with their practice, method and medicines. But in observing them I noted much practice but little theory: therefore not contenting myself to have but one string to my bow, lest a time of night might overtake me wanting oile in my lamp and labouring to be as cunning in knowledge of the reason as perfect in practice upon the occasion, I addressed myself to the study of divers best approved authors in which reading I found many windows that let in the light of reason to make my practice the more judicious so I also met with some shops full of false lights that gave a gayer colour and a finer thread to many medicines than they merited." Banister having thus fitted himself for the practice of his profession was dismayed by "the boundless boldness of many women who for lack of learning cannot be acquainted with the theoricke part and yet dare venture on the practicke. I believe that scarce three of thirteen hundred can define or describe the names and natures of the hundred and thirteen diseases of the eye, yet having snatched up some one or two medicines only they think themselves armed against all diseases. Let these women therefore either apply themselves to learn the grounds of their practice or leave their practice to them that are better grounded, that so they may cease by their ignorance to make them blind, that by our art might be made to see. Many have come from these to me lighted more by sorrow than by sight with their eyes full of tears but empty of optic humours. And yet these firebrands that smoke and choke folks eyes out can take hens, chickens, and such reasonable rewards for their unreasonable wrongs: let all that love their eyes make better use of their sight before night come than to seek out such empty empiricks that will make them with grief go groping home in the midday sun. But in this general reprehension I would not be accounted to cry out on all women without exception that are addicted to Chirurgerie for I have known some whose worth and wisdom might be paralleled with principal men whose cures were attended with due care and ended with true charity, as the right religious and virtuous Lady Mildmay of Apthorpe in the county of Northampton who has herself good judgment in many things: yet when the poor came home to her for help (for on such people she did plan her practice) in cases of physic, she would use the approbation of a physician; in chirurgerie the aid of a chirurgian: and for the eyes the assistance of myself. With her in the like regard may be reckoned good Mistress Dorington of Godstowe who would ever refuse to meddle with the rich lest art should go unmaintained, and because the needy should receive no hurt by her she always adjoined the advice of the artist with her own endeavours. These are the Black Swannes of this silver age. I wish the rest would look on these as patterns of goodness, and then they should soon see the true way to those good deeds they seek to get a good name by."

And here we will leave Master Richard Banister in the hope that at a future time we may be able to show the advances he made in his art.

